# Mutual information estimation reveals global associations between stimuli and biological processes

**DOI:** 10.1186/1471-2105-10-S1-S52

**Published:** 2009-01-30

**Authors:** Taiji Suzuki, Masashi Sugiyama, Takafumi Kanamori, Jun Sese

**Affiliations:** 1 Department of Mathematical Informatics, The University of Tokyo, 7-3-1 Hongo, Bunkyo-ku, Tokyo 113-8656, Japan; 2 Department of Computer Science, Tokyo Institute of Technology, 2-12-1 O-okayama, Meguro-ku, Tokyo 152-8552, Japan; 3 Department of Computer Science and Mathematical Informatics, Nagoya University, Furocho, Chikusaku, Nagoya 464-8603, Japan; 4 Department of Information Science, Ochanomizu University, 2-1-1 Ohtsuka, Bunkyo-ku, Tokyo 112-8610, Japan

## Abstract

**Background:**

Although microarray gene expression analysis has become popular, it remains difficult to interpret the biological changes caused by stimuli or variation of conditions. Clustering of genes and associating each group with biological functions are often used methods. However, such methods only detect partial changes within cell processes. Herein, we propose a method for discovering global changes within a cell by associating observed conditions of gene expression with gene functions.

**Results:**

To elucidate the association, we introduce a novel feature selection method called *Least-Squares Mutual Information (LSMI)*, which computes mutual information without density estimaion, and therefore LSMI can detect nonlinear associations within a cell. We demonstrate the effectiveness of LSMI through comparison with existing methods. The results of the application to yeast microarray datasets reveal that non-natural stimuli affect various biological processes, whereas others are no significant relation to specific cell processes. Furthermore, we discover that biological processes can be categorized into four types according to the responses of various stimuli: DNA/RNA metabolism, gene expression, protein metabolism, and protein localization.

**Conclusion:**

We proposed a novel feature selection method called LSMI, and applied LSMI to mining the association between conditions of yeast and biological processes through microarray datasets. In fact, LSMI allows us to elucidate the global organization of cellular process control.

## Background

Advances in microarray technologies enable us to explore the comprehensive dynamics of transcription within a cell. The current problem is to extract useful information from a massive dataset. The primarily used approach is clustering. Cluster analysis reveals variations of gene expression and reduces the complexity of large datasets. However, additional methods are necessary to associate genes in each cluster with genetic function using GO term finder [1], or to understand stimuli related to specific cellular status.

However, these clustering-association strategies cannot detect global cell status changes because of the division of clusters. Some stimuli activate a specific pathway, although others might change overall cellular processes. Understanding the effect of stimuli in cellular processes directly, in this paper, we introduce a novel feature selection method called Least-Squares Mutual Information (LSMI), which selects features using mutual information without density estimation. Mutual information has been utilized to measure distances between gene expressions [2]. To compute the mutual information in existing methods, density estimation or discritization is required. However, the estimation of gene expression is difficult because we have little knowledge about density function of gene expression profile. LSMI offers an analytic-form solution and avoid the estimation.

Feature selection techniques are often used in gene expression analysis [3]. Actually, LSMI has three advantages compared to existing methods: capability of avoiding density estimation which is known to be a hard problem [4], availability of model selection, and freedom from a strong model assumption. To evaluate the reliability of ranked features using LSMI, we compare receiver operating characteristic (ROC) curves [5] to those of existing methods: kernel density estimation (KDE) [6,7], k-nearest neighbor (KNN) [8], Edgeworth expansion (EDGE) [9], and Pearson correlation coefficient (PCC). Thereby, we certify that our method has better performance than the existing methods in prediction of gene functions about biological processes. This fact implies that features selected using our method reflect biological processes.

Using the ranked features, we illustrate the associations between stimuli and biological processes according to gene expressions. Results show that stimuli damage essential processes within a cell, causing association with some cellular processes. From the response to stimuli, biological processes are divisible into four categories: DNA/RNA metabolic processes, gene expression, protein metabolic processes, and protein localization.

## Results

### Approach – mutual information detection

In this study, we detect underlying dependencies between gene expressions obtained by groups of stimuli and gene functions. The dependencies are studied in various machine learning problems such as feature selection [10,11] and independent component analysis [12]. Although classical correlation analysis would be useful for these problems, it cannot detect nonlinear dependencies with no correlation. On the other hand, *mutual information *(MI), which plays an important role in information theory [13], enables us to detect general nonlinear dependencies. Let ***x ***and ***y ***be a set of gene expressions and a set of known gene functions. A variant of MI based on the squared loss is defined by

(1) $$\begin{array}{r} I_{s}(X,Y):=\iint {\left(\frac{p_{\text{xy}}(x,y)}{p_{\text{x}}(x)p_{\text{y}}(y)}-1\right)}^{2}\\ \times p_{\text{x}}(x)p_{\text{y}}(y)dxdy. \end{array}$$

Note that *I*_*s *_vanishes if and only if ***x ***and ***y ***are independent. The use of MI allows us to detect no correlation stimulus with a specific gene function or process.

Estimating MI is known to be a difficult problem in practice [8,9,11]. Herein, we propose LSMI, which does not involve density estimation but directly models the *density ratio*:

$$w(x,y):=\frac{p_{\text{xy}}(x,y)}{p_{\text{x}}(x)p_{\text{y}}(y)}.$$

Given a density ratio estimator $\hat{w}$(***x***, ***y***), squared loss MI can be simply estimated by

$${\hat{I}}_{s}(X,Y)=\frac{1}{n^{2}}\sum_{i,j=1}^{n}{\left(\hat{w}(x_{i},y_{j})-1\right)}^{2}.$$

Mathematical definitions related to LSMI are provided in the Methods section. LSMI offers an analytic-form solution, which allows us to estimate MI in a computationally very efficiently manner. It is noteworthy that ***x ***includes a multi-dimensional vector. In fact, LSMI can handle a group of stimuli, although generic correlation indices such as Pearson correlation between parameters and target value are calculated independently. Therefore, we can elucidate which type of stimulus has no dependency to biological processes using LSMI.

### Datasets and feature selection

In this section, we first prepare datasets to show the association between stimuli and biological process, and introduce feature selection using the datasets.

#### Biological process

We compute mutual information between gene expression values grouped by stimuli and class of genes' biological processes. As the class, we use biological process terms in Gene Ontology (GO) categorization [14]. We select GO terms associated with more than 800 and less than 2,000 genes because terms having a small number of genes only describe a fraction of the cell status, whereas terms having a large number of genes indicate functions associated with almost all genes in yeast. Actually, GO has a directed acyclic graph (DAG) structure, and each term has child terms. The GO terms are classified into three categories; we use only biological process terms to identify the changes within a cell. Using this method, we select 12 GO terms.

#### Gene expression profiles

The gene expression profile is the best comprehensive dataset to associate stimuli and biological processes. We use two different microarray datasets. One is of 173 microarray data under stress conditions of various types [15]. We categorize the 173 stress conditions into 29 groups based on the type of condition such as heat shock, oxidizing condition, etc. The other is of 300 microarray data under gene-mutated conditions [16]. We categorize the genes into 146 groups based on associated GO terms. We use only the GO terms which are associated with 1,500 genes or fewer. We also use child terms on a GO layered structure if the term has more than 1,200 genes. When one gene belongs to multiple GO terms, we classify the gene into the the classification whose number of associated genes is smallest. In both profiles, we remove genes whose expression values are obtained from fewer than 30% of all observed conditions. All missing values are filled out by the average of all the expression values.

#### Feature selection using LSMI

We use a novel feature selection method called LSMI, which is based on MI, to associate stimuli with cellular processes. Here we consider the *forward *feature-group addition strategy, i.e., a feature-group score between each input feature-group and output cellular process is computed. The top *m *feature-groups are used for training a classifier. We predict 12 GO terms independently. We randomly choose 500 genes from among 6, 116 genes on the stress condition dataset for feature-group selection and for training a classifier; the rest are used for evaluating the generalization performance. For using the gene-mutated expression dataset, we select 500 genes from among 6, 210 genes. We repeat this trial 10 times. For classification, we use a Gaussian kernel support vector machine (GK-SVM) [4], where the kernel width is set at the median distance among all samples and the regularization parameter is fixed at *C *= 10. We explain the efficiency of feature selection of LSMI in the Discussion section.

## Results

**Figure 1:**
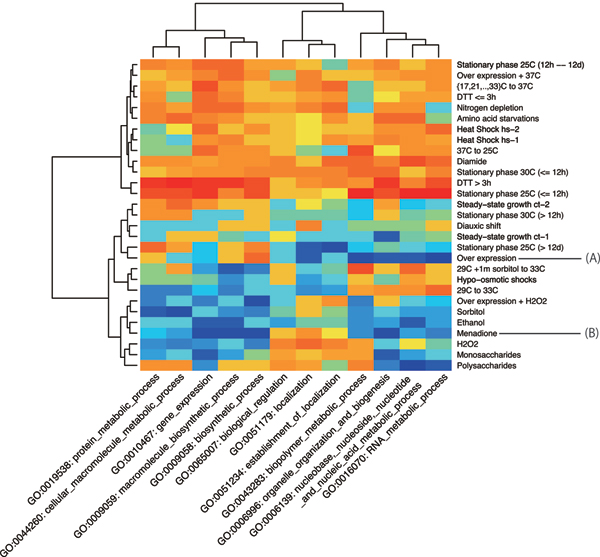
**Stress conditions versus biological processes**. Matrix of stress conditions (rows) versus biological processes (columns). Red cells have higher correlation.

The association between stress conditions and biological processes in GO terms is shown in Fig. 1. Each row and column respectively indicate a group of conditions and a GO term. Row and column dendrograms are clustering results by the Ward method according to cell values. Each cell contains an average ranking over 10 trials by LSMI. The red cell denotes that the parameter has a higher rank; that is, the parameter has association with the target GO term. A blue cell denotes that the parameter has a lower rank.

As shown in this figure, conditions are divided into two groups. Almost all conditions in the upper cluster have higher rank, whereas those in a lower cluster have higher rank only under specific conditions. The conditions in the upper cluster include strong heat shocks, dithiothreitol (DTT) exposure, nitrogen depletion, and diamide treatments, which are non-natural conditions. The result reveals that non-natural conditions change overall cellular processes.

The GO term clusters are divided into three groups: DNA/RNA metabolism (right), localization of protein (middle), and others (left). The leftmost cluster contains bio synthesis, gene expression process, and protein metabolic process. From this figure, nucleic acid metabolism processes are inferred to be independent from amino acid metabolism processes. We will confirm the independence and consider the division of clusters by using other dataset later.

We herein investigate the details of difference among DNA metabolic process, protein metabolic process and localization of proteins. Under an overexpression condition indicated by sign (A) in Fig. 1, DNA/RNA metabolisms show no correlation with expressions of genes belonging to over-expression genes. This finding of no correlation is one advantage of LSMI. The menadione (vitamin K) exposure condition indicated by (B) in Fig. 1 is associated with localization of proteins. Menadione supplementation causes high toxicity; such toxicity might result from the violation of protein localizations.

**Figure 2:**
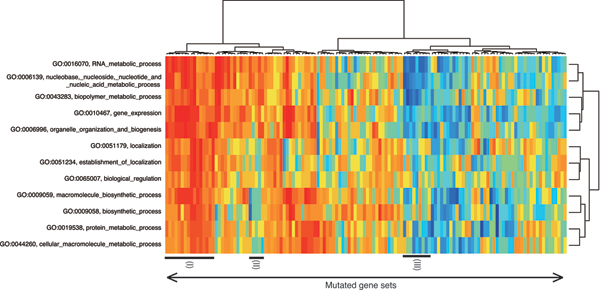
**Mutated gene groups versus biological processes**. Overview: a matrix of mutated gene groups (rows) versus biological processes (columns).

Next, we compute the association using expressions of gene mutants. The results are shown in Fig. 2. The stimulus can be categorized into two parts: high association under almost all processes and under particular conditions. The division is the same because of stress condition associations. The GO terms also categorize three parts: DNA/RNA metabolic processes, protein metabolic processes, and localization. In this experiment, GO terms "gene expression" (GO:0010467) and "organelle organization and biogenesis" (GO:0006996) are in the DNA/RNA metabolic process cluster, although they are classified in protein metabolic processes cluster under stress conditions in Fig. 1. Because the both divisions are close to ancestor division, we can conclude that the cluster about gene expression exists. From these results, GO terms are divisible into four categories: DNA/RNA metabolic process, protein metabolic process, localization, and gene expression.

**Figure 3:**
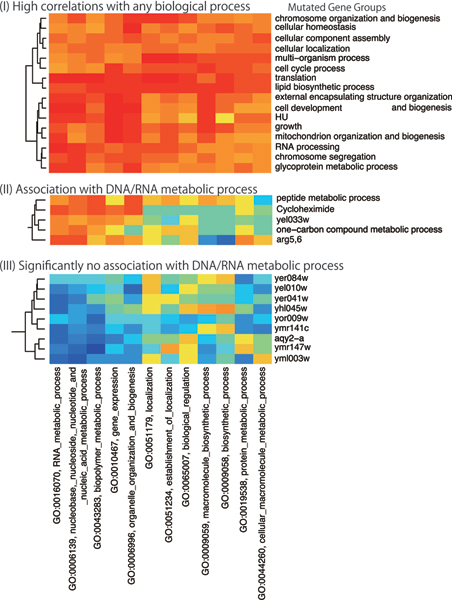
Submatrices of Figure 2.

In Fig. 3, we present details of three clusters in Fig. 2. In fact, Fig. 3(I) presents a cluster whose members are correlated with any biological process. Furthermore, the functions of the mutated genes are essential processes for living cells, such as cellular localization, cell cycle, and growth. This result might indicate that the upper half stimulus in Fig. 1 destroys the functions of these essential genes. Furthermore, Fig. 3(II) includes the groups of genes associated with DNA/RNA metabolic processes. In this cluster, YEL033W/MTC1 is a gene with unknown function and is predicted to have a metabolic role using protein-protein interaction [17]. Our clustering result indicates that YEL033W would have some relation with metabolism, especially methylation (methylation is an important part of the one-carbon compound metabolic process). We show genes which have no significant association with DNA/RNA metabolic processes in Fig. 3(III). In the cluster, all genes except AQY2 are of unknown function. No correlation clusters cannot be found by existing methods. Our result might provide clues to elucidate these genes' functions.

## Discussion

A common analytical flow of the expression data is first clustering and then associating clusters with GO terms or pathways. Although clustering reduces the complexity of large datasets, the strategy might fail to detect changes of entire genes within a cell such as metabolic processes.

To interpret such gene expression changes, gene set enrichment analysis [18] has been proposed. This method treats microarrays independently. Therefore, housekeeping genes are often ranked highly. When gene expressions under various conditions are available, our method would show us the better changes of cellular processes because of the comparison between groups of conditions. The module map [19] gives a global association between a set of genes and a set of conditions. However, this method requires important changes of gene expressions because it uses hypergeometric distributions to compute correlations. Our correlation index is based on MI. Therefore, we can detect nonlinear dependencies with no correlation. An example is depicted in Fig. 3(III).

The characteristics of LSMI and existing MI estimators are presented in Table 1. Detail comparisons are described in the Methods section. The *kernel density estimator *(KDE) [6,7] is distribution-free. Model selection is possible by likelihood cross-validation (LCV). However, a hard task of density estimation is involved. Estimation of the *entropies *using *k*-nearest neighbor (KNN) samples [8] is distribution-free and does not involve density estimation directly. However, no model selection method exists for determining the number of nearest neighbors. Edgeworth expansion (EDGE) [9] does not involve density estimation or any tuning parameters. However, it is based on the assumption that the target distribution is close to the normal distribution. On the other hand, LSMI is distribution-free; it involves no density estimation, and model selection is possible by cross-validation (CV). Therefore, LSMI overcomes limitations of the existing approaches. Within a cell, most processes have a nonlinear relation such as enzyme effects and feedback loops. The lack of one advantage might cause difficulty of application to biological datasets. By virtue of these advantages, LSMI can detect correlation or independence between features of complex cellular processes.

**Table 1:** Relation between existing and proposed MI estimators. If the order of the Edgeworth expansion is regarded as a tuning parameter, model selection of EDGE is expected to be 'Not available'.

	Density estimation	Model selection	Distribution
KDE	Involved	**Available**	**Free**

KNN	**Not involved**	Not available	**Free**

EDGE	**Not involved**	**Not necessary**	Nearly normal

LSMI	**Not involved**	**Available**	**Free**

**Figure 4:**
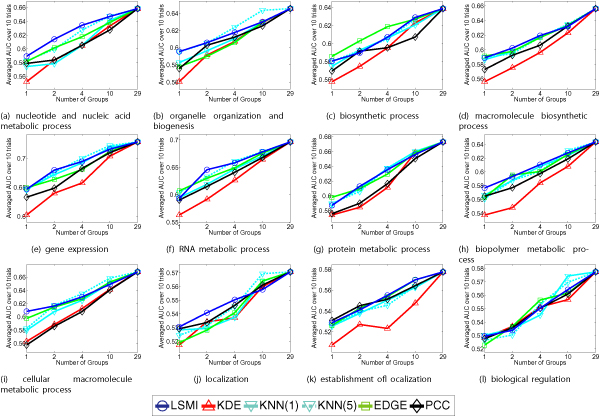
**Classification error**. Classification error against the number of feature groups for the yeast cell datasets.

To investigate the efficiency of feature selection, we compare areas under the curve (AUCs) with LSMI (CV), KDE(LCV), KNN(*k*) for *k *= 1, 5, EDGE, and PCC. Details of these methods are described in the Methods section. Fig. 4 depicts AUCs for 12 GO term classifications. The *x*-axis shows the number of stimulus groups used for the prediction. The *y*-axis means averaged AUC over 10 trials, where AUCs are calculated as the area under the receiver operating characteristic (ROC) curve, which is often used for diagnostic tests. Each figure shows AUC curves calculated using the six methods.

In the AUC figures, the higher curves represent better predictions. For example, Fig. 4(a) shows that LSMI is the highest position, which means that LSMI achieves the best performance among the six methods. In Figs. 4(b) and 4(d), KNN(1) and KNN(5), which are denoted by the light blue and dotted light blue lines, have the best performance. However, in Figs. 4(i), (j) and 4(l), averaged AUCs of KNN using numerous groups are high, whereas the AUCs using small and few groups are low. No systematic model selection strategies exist for KNN and therefore KNN would be unreliable in practice. Fig. 4(c) depicts that EDGE, which is indicated by the light green line, has the highest AUC. In fact, EDGE presumes the normal distribution. Consequently, it works well only on a few datasets. From these figures, LSMI indicated by the blue line appears to be the best feature selection method.

## Conclusion

We provided a global view of the associations between stimuli and changes of biological processes based on gene expression profiles. The association is generally difficult to use for making models because of nonlinear correlation. To cope with this problem, we introduced a novel feature selection method called *LSMI*, which uses MI and can be computed efficiently. In comparison to other feature selection methods, LSMI showed better AUCs in prediction of biological process functions. Consequently, our feature selection results would be more reliable than those obtained using the other methods. We calculated the association between stimuli and GO biological process terms using gene expression profiles. The result revealed that the stimuli are categorized into four types: related to DNA/RNA metabolic process, gene expression, protein metabolic process, and protein localization. LSMI enabled us to reveal the global regulation of cellular processes from comprehensive transcription datasets.

## Methods

### Mutual information estimation

A naive approach to estimating MI is to use a KDE [6,7], i.e., the densities *p*_xy_(***x***, ***y***), *p*_x_(***x***), and *p*_y_(***y***) are separately estimated from samples and the estimated densities are used for computing MI. The band-width of the kernel functions could be optimized based on likelihood cross-validation (LCV) [20], so there remains no open tuning parameter in this approach. However, density estimation is known to be a hard problem [4] and therefore the KDE-based method may not be so effective in practice.

An alternative method involves estimation of entropies using KNN. The KNN-based approach was shown to perform better than KDE [21], given that the number *k *is chosen appropriately – a small (large) *k *yields an estimator with small (large) bias and large (small) variance. However, appropriately determining the value of *k *is not straightforward in the context of MI estimation.

Here, we propose a new MI estimator that can overcome the limitations of the existing approaches. Our method, which we call Least-Squares Mutual Information (LSMI), does not involve density estimation and directly models the *density ratio*:

(2) $$w(x,y):=\frac{p_{\text{xy}}(x,y)}{p_{\text{x}}(x)p_{\text{y}}(y)}.$$

The solution of LSMI can be computed by simply solving a system of linear equations. Therefore, LSMI is computationally very efficient. Furthermore, a variant of cross-validation (CV) is available for model selection, so the values of tuning parameters such as the regularization parameter and the kernel width can be adaptively determined in an objective manner.

### A new MI estimator

In this section, we formulate the MI inference problem as density ratio estimation and propose a new method of estimating the density ratio.

#### MI inference via density ratio estimation

Let $D_{\text{X}}(\subset {\mathbb{R}}^{d_{\text{x}}})$ and $D_{\text{Y}}(\subset {\mathbb{R}}^{d_{\text{y}}})$ be the data domains and suppose we are given *n *independent and identically distributed (i.i.d.) paired samples

$${\left\{(x_{i},y_{i})|x_{i}\in D_{\text{X}},y_{i}\in D_{\text{Y}}\right\}}_{i=1}^{n}$$

drawn from a joint distribution with density *p*_xy_(***x***, ***y***). Let us denote the marginal densities of ***x***_*i *_and ***y***_*i *_by *p*_x_(***x***) and *p*_y_(***y***), respectively. The goal is to estimate squared-loss MI defined by Eq.(1).

Our key constraint is that we want to avoid density estimation when estimating MI. To this end, we estimate the *density ratio w*(***x***, ***y***) defined by Eq.(2). Given a density ratio estimator $\hat{w}$(***x***, ***y***), MI can be simply estimated by

$${\hat{I}}_{s}(X,Y)=\frac{1}{n^{2}}\sum_{i,j=1}^{n}{\left(\hat{w}(x_{i},y_{j})-1\right)}^{2}.$$

We model the density ratio function *w*(***x***, ***y***) by the following linear model:

$${\hat{w}}_{\alpha }(x,y):=\alpha^{⊤}\varphi (x,y),$$

where ***α ***= (*α*_1_, *α*_2_, ..., *α*_*b*_)^*⊤ *^are parameters to be learned from samples, ^*⊤ *^denotes the transpose of a matrix or a vector, and

***φ***(***x***, ***y***) = (*φ*_1_(**x**,**y**), *φ*_2_(**x**, **y**), ..., *φ*_*b*_(**x**,**y**))^*⊤*^

are basis functions such that

$$\begin{array}{ccc} \varphi (x,y)\geq 0_{b} & forall & (x,y)\in D_{\text{X}}\times D_{\text{Y}}. \end{array}$$

**0**_*b *_denotes the *b*-dimensional vector with all zeros. Note that ***φ***(***x***, ***y***) could be dependent on the samples ${\left\{x_{i},y_{i}\right\}}_{i=1}^{n}$, i.e., *kernel *models are also allowed. We explain how the basis functions ***φ***(***x***, ***y***) are chosen in the later section.

#### A least-squares approach to direct density ratio estimation

We determine the parameter ***α ***in the model ${\hat{w}}_{\alpha }$(***x***, ***y***) so that the following squared error *J*_0 _is minimized:

$$\begin{array}{c} J_{0}(\alpha ):=\frac{1}{2}\iint {\left({\hat{w}}_{\alpha }(x,y)-w(x,y)\right)}^{2}p_{\text{x}}(x)p_{\text{y}}(y)dxdy\\ =\frac{1}{2}\iint {\hat{w}}_{\alpha }{\left(x,y\right)}^{2}p_{\text{x}}(x)p_{\text{y}}(y)dxdy\\ -\iint {\hat{w}}_{\alpha }(x,y)p_{\text{xy}}(x,y)dxdy+C, \end{array}$$

where $C=\frac{1}{2}\iint w(x,y)p_{\text{xy}}(x,y)dxdy$ is a constant and therefore can be safely ignored. Let us denote the first two terms by *J*:

$$J(\alpha ):=J_{0}(\alpha )-C=\frac{1}{2}\alpha^{⊤}H\alpha -h^{⊤}\alpha ,$$

where

$$\begin{array}{c} H:=\iint \varphi (x,y)\varphi {\left(x,y\right)}^{⊤}p_{\text{x}}(x)p_{\text{y}}(y)dxdy,\\ h:=\iint \varphi (x,y)p_{\text{xy}}(x,y)dxdy. \end{array}$$

Approximating the expectations in ***H ***and ***h ***by empirical averages, we obtain the following optimization problem:

(3) $$\tilde{\alpha }:=\underset{\alpha \in {\mathbb{R}}^{b}}{\arg\min}\left[\frac{1}{2}\alpha^{⊤}\hat{H}\alpha -{\hat{h}}^{⊤}\alpha +\lambda \alpha^{⊤}\alpha \right],$$

where we included a regularization term *λ****α***^*⊤*^***α ***and

$$\begin{array}{c} \hat{H}:=\frac{1}{n^{2}}\sum_{i,j=1}^{n}\varphi (x_{i},y_{j})\varphi {\left(x_{i},y_{j}\right)}^{⊤},\\ \hat{h}:=\frac{1}{n}\sum_{i=1}^{n}\varphi (x_{i},y_{i}). \end{array}$$

Differentiating the objective function (3) with respect to ***a ***and equating it to zero, we can obtain an analytic-form solution:

$$\tilde{\alpha }={\left(\hat{H}+\lambda I_{b}\right)}^{-1}\hat{h},$$

where ***I***_*b *_is the *b*-dimensional identity matrix.

We call the above method *Least-Squares Mutual Information (LSMI)*. Thanks to the analytic-form solution, the LSMI solution can be computed very efficiently.

#### Convergence bound

Here, we show a non-parametric convergence rate of the solution of the optimization problem (3).

Let G be a general set of functions on $D_{X}\times D_{Y}$. For a function *g *(∈ G), let us consider a non-negative function *R*(*g*) such that

$$\underset{x,y}{\sup}[g(x,y)\leq R(g)].$$

Then the problem (3) can be generalized as

$$\hat{w}:=\underset{g\in G}{\arg\min}\left[\frac{1}{2n^{2}}\sum_{i,j=1}^{n}g_{i,j}^{2}-\frac{1}{n}\sum_{i=1}^{n}g_{i,i}+\lambda_{n}R{\left(g\right)}^{2}\right],$$

where *g*_*i*,*j *_:= *g*(***x***_*i*_, ***y***_*j*_). We assume that the true density ratio function *w*(***x***, ***y***) is contained in model G and satisfies

*w*(***x*, *y***) <*M*_0 _for all (***x*, *y***) ∈ *D*_*X *_× *D*_*Y*_.

We also assume that there exists *γ *(0 <*γ *< 2) such that

$$H_{\left[\right]}(G_{M},\epsilon ,L_{2}(p_{X}p_{Y}))=O({\left(M/\epsilon \right)}^{\gamma }),$$

where

$$G_{M}:=\{g\in G|R(g)\leq M\}$$

and $H_{\left[\right]}$ is the *bracketing entropy *of $G_{M}$ with respect to the *L*_2_(*p*_x_*p*_y_)-norm [22,23]. This means the function class G is not too much complex.

Then we have the following theorem. Its proof is omitted due to lack of space.

**Theorem 1 ***Under the above setting, if λ*_*n *_→ 0 *and *$\lambda_{n}^{-1}$ = *o*(*n*^2/(2+*γ*)^) *then*

$${\left\|\hat{w}-w\right\|}_{2}=O_{p}(\lambda_{n}^{1/2}),$$

*where *||·||_2 _*means the L*_2_(*p*_x_*p*_y_)-*norm and *$O_{p}$*denotes the asymptotic order in probability*.

This theorem is closely related to [24,25]. [24] considers least squares estimators for nonparametric regression, and related topics can be found in Section 10 of [23].

#### CV for model selection and basis function design

The performance of LSMI depends on the choice of the model, i.e., the basis functions ***φ***(***x***, ***y***) and the regularization parameter *λ*. Here we show that model selection can be carried out based on a variant of CV.

First, the samples ${\left\{z_{i}|z_{i}=(x_{i},y_{i})\right\}}_{i=1}^{n}$ are divided into *K *disjoint subsets ${\left\{Z_{k}\right\}}_{k=1}^{K}$. Then a density ratio estimator ${\hat{w}}_{k}$(***x***, ***y***) is obtained using {*Ƶ*_*j*_}_*j *≠ *k *_and the cost J is approximated using the held-out samples *Ƶ*_*k *_as

$${\hat{J}}_{k}^{\left(K-\text{CV}\right)}=\sum_{x^{\prime },y^{\prime }\in Z_{k}}\frac{{\hat{w}}_{k}{\left(x^{\prime },y^{\prime }\right)}^{2}}{2n_{k}^{2}}-\sum_{(x^{\prime },y^{\prime })\in Z_{k}}\frac{{\hat{w}}_{k}(x^{\prime },y^{\prime })}{n_{k}},$$

where *n*_*k *_is the number of pairs in the set *Ƶ*_*k*_. $\sum_{x^{\prime },y^{\prime }\in Z_{k}}$ is the summation over all combinations of ***x' ***and ***y' ***(i.e., $n_{k}^{2}$ terms), while $\sum_{(x^{\prime },y^{\prime })\in Z_{k}}$ is the summation over all pairs (***x'***, ***y'***) (i.e., *n*_*k *_terms). This procedure is repeated for *k *= 1, 2, ..., *K *and its average ${\hat{J}}^{\left(K-\text{CV}\right)}$ is used as an estimate of *J*:

$${\hat{J}}^{\left(K-\text{CV}\right)}=\frac{1}{K}\sum_{k=1}^{K}{\hat{J}}_{r}^{\left(K-\text{CV}\right)}.$$

We can show that ${\hat{J}}^{\left(K-\text{CV}\right)}$ is an almost unbiased estimate of the true cost *J*, where the 'almost'-ness comes from the fact that the number of samples is reduced in the CV procedure due to data splitting [4]. A good model may be chosen by CV, given that a family of promising model candidates is prepared. As model candidates, we propose using a Gaussian kernel model:

$$\varphi_{\ell }\left(x,y\right)=\exp\left(-\frac{{\left\|x-u_{\ell }\right\|}^{2}}{2\sigma^{2}}\right)\delta \left(y=v_{\ell }\right),$$

Where

$${\left\{(u_{\ell },v_{\ell })\right\}}_{\ell =1}^{b}$$

are 'center' points randomly chosen from

$${\left\{(x_{i},y_{i})\right\}}_{i=1}^{n}.$$

*δ*(***y ***= ***v***_ℓ_) is a indicator function, which is 1 if ***y ***= ***v***_ℓ _and 0 otherwise.

In the experiments, we fix the number of basis functions at

*b *= min(100, *n*),

and choose the Gaussian width *σ *and the regularization parameter *λ *by CV with grid search.

### Relation to existing methods

In this section, we discuss the characteristics of existing and proposed approaches.

Kernel density estimator (KDE)

KDE [6,7] is a non-parametric technique to estimate a probability density function *p*(***x***) from its i.i.d. samples ${\left\{x_{i}\right\}}_{i=1}^{n}$. For the Gaussian kernel, KDE is expressed as

$$\hat{p}(x)=\frac{1}{n{\left(2\pi \sigma^{2}\right)}^{d/2}}\sum_{i=1}^{n}\exp\left(-\frac{{\left\|x-x_{i}\right\|}^{2}}{2\sigma^{2}}\right).$$

The performance of KDE depends on the choice of the kernel width *σ *and it can be optimized by *likelihood CV *as follows [20]: First, divide the samples ${\left\{x_{i}\right\}}_{i=1}^{n}$ into *K *disjoint subsets ${\left\{X_{k}\right\}}_{n=1}^{K}$. Then obtain a density estimate ${\hat{p}}_{X_{k}}$(***x***) from ${\left\{X_{j}\right\}}_{j\neq k}$ and compute its hold-out log-likelihood for $X_{k}$:

$$\frac{1}{\left|X_{k}\right|}\sum_{x\in X_{k}}\log{\hat{p}}_{X_{k}}\left(x\right).$$

This procedure is repeated for *k *= 1, 2, ..., *K *and choose the value of *σ *such that the average of the hold-out log-likelihood over all *k *is maximized. Note that the average hold-out log-likelihood is an almost unbiased estimate of the Kullback-Leibler divergence from *p*(***x***) to $\hat{p}$(***x***), up to an irrelevant constant.

Based on KDE, MI can be approximated by separately estimating the densities *p*_xy_(***x***, ***y***), *p*_x_(***x***) and *p*_y_(***y***) using ${\left\{x_{i},y_{i}\right\}}_{i=1}^{n}$. However, density estimation is known to be a hard problem and therefore the KDE-based approach may not be so effective in practice.

#### k-nearest neighbor method (KNN)

Let $N_{k}$(*i*) be the set of *k*-nearest neighbor samples of (***x***_*i*_, ***y***_*i*_), and let

$$\begin{array}{l} \epsilon_{\text{x}}\left(i\right):=\max\left\{\left\|x_{i}-x_{i^{\prime }}\right\||\left(x_{i}-y_{i^{\prime }}\right)\in N_{k}\left(i\right)\right\},\\ \epsilon_{\text{y}}\left(i\right):=\max\left\{\left\|y_{i}-y_{i^{\prime }}\right\||\left(x_{i^{\prime }}-y_{i^{\prime }}\right)\in N_{k}\left(i\right)\right\},\\ n_{\text{x}}\left(i\right):=\#\left\{z_{i^{\prime }}|\left\|x_{i}-x_{i^{\prime }}\right\|\leq \epsilon_{\text{x}}\left(i\right)\right\},\\ n_{\text{y}}\left(i\right):=\#\left\{z_{i^{\prime }}|\left\|y_{i}-y_{i^{\prime }}\right\|\leq \epsilon_{\text{y}}\left(i\right)\right\}. \end{array}$$

Then the KNN-based MI estimator is given as follows 8:

$$\begin{array}{c} \hat{I}\left(X,Y\right)=\psi \left(k\right)+\psi \left(n\right)-\frac{1}{k}\\ -\frac{1}{n}\sum_{i=1}^{n}\left[\psi \left(n_{x}\left(i\right)\right)+\psi \left(n_{y}\left(i\right)\right)\right], \end{array}$$

where *ψ *is the *digamma *function.

A practical drawback of the KNN-based approach is that the estimation accuracy depends on the value of *k *and there seems no systematic strategy to choose the value of *k *appropriately.

#### Edgeworth expansion (EDGE)

MI can be expressed in terms of the entropies as

*I*(*X*, *Y*) = *H*(*X*) + *H*(*Y*) - *H*(*X*, *Y*),

where *H*(*X*) denotes the entropy of *X*:

$$H\left(X\right):=-\int p_{\text{x}}\left(x\right)\log p_{\text{x}}\left(x\right)dx.$$

Thus MI can be approximated if the entropies above are estimated.

In the paper [9], an entropy approximation method based on the *Edgeworth expansion *is proposed, where the entropy of a distribution is approximated by that of the normal distribution and some additional higher-order correction terms. More specifically, for a *d*-dimensional distribution, the entropy is approximated by

$$\begin{array}{c} H\approx H_{\text{normal}}-\frac{1}{12}\sum_{i=1}^{d}\kappa_{i,i,i}^{2}-\frac{1}{4}\sum_{i,j=1,i\neq j}^{d}\kappa_{i,i,j}^{2}\\ -\frac{1}{72}\sum_{i,j,k=1,i<j<k}^{d}\kappa_{i,j,k}^{2}, \end{array}$$

where *H*_normal _is the entropy of the normal distribution with covariance matrix equal to the target distribution and κ_*i*,*j*,*k *_(1 ≤ *i*, *j*, *k *≤ *d*) is the standardized third cumulant of the target distribution. In practice, all the cumulants are estimated from samples.

If the underlying distribution is close to the normal distribution, the above approximation is quite accurate and the EDGE method works very well. However, if the distribution is far from the normal distribution, the approximation error gets large and therefore the EDGE method may be unreliable. In principle, it is possible to include the fourth and even higher cumulants for further reducing the estimation bias. However, this in turn increases the estimation variance; the expansion up to the third cumulants would be reasonable.

## Competing interests

The authors declare that they have no competing interests.

## Authors' contributions

TS developed the method, implemented the algorithm and wrote the manuscript. MS and TK discussed the method and revised the manuscript. JS discussed the method, interpreted the results and wrote the manuscript.
